# Effects of Ethanol Exposure and Withdrawal on Neuronal Morphology in the Agranular Insular and Prelimbic Cortices: Relationship with Withdrawal-Related Structural Plasticity in the Nucleus Accumbens

**DOI:** 10.3390/brainsci9080180

**Published:** 2019-07-27

**Authors:** Madeline E. Frost, Veronica L. Peterson, Clark W. Bird, Brian McCool, Derek A. Hamilton

**Affiliations:** 1 Department of Psychiatry and Behavioral Sciences, University of New Mexico, Albuquerque, NM 87131, USA; 2 Teagasc Food Bioscience Research Centre, Carlow R93 XE12, Ireland; 3 Department of Neurosciences, University of New Mexico, Albuquerque, NM 87131, USA; 4 Department of Physiology & Pharmacology, Wake Forest University School of Medicine, Winston-Salem, NC 27109, USA; 5 Department of Psychology, University of New Mexico, Albuquerque, NM 87131, USA

**Keywords:** alcohol, frontal cortex, withdrawal, plasticity, Golgi, dendritic morphology, nucleus accumbens, chronic intermittent ethanol exposure

## Abstract

The present study investigated the effects of chronic intermittent ethanol exposure and withdrawal on dendritic morphology and spine density in the agranular insular and prelimbic cortices. Adult male Sprague–Dawley rats were passively exposed to vaporized ethanol (~37 mg/L; 12 h/day) or air (control) for ten consecutive days. Dendritic length, branching, and spine density were quantified in layer II/III pyramidal neurons 24 hours or seven days following the final ethanol exposure. Compared to unexposed control animals there were structural alterations on neurons in the prelimbic cortex, and to a lesser extent the agranular insular cortex. The most prominent ethanol-related differences were the transient increases in dendritic length and branching in prelimbic neurons at 24 h post-cessation, and increased mushroom-shaped spines at seven days post-cessation. The results obtained in the prelimbic cortex are the opposite of those previously reported in the nucleus accumbens core (Peterson, et al. 2015), suggesting that these regions undergo distinct functional adaptations following ethanol exposure and withdrawal.

## 1. Introduction

Alcohol exposure and withdrawal induce robust modifications in dendritic length, branching, and spine density on neurons within circuits implicated in drug addiction and reward [1,2,3,4,5,6]. Such structural modifications represent alterations in the space available for synaptic connections and are among the major neurobiological adaptations by which experience alters the brain in the service of future behavior [7,8,9]. Consequently, modifications in dendritic morphology following drug exposure and cessation may contribute to the constellation of behavioral and cognitive alterations observed during withdrawal [10,11,12,13,14]. The quantification of such structural changes in neurons can aid in the identification of brain regions and circuits involved in these outcomes. 

The goal of the present study was to evaluate the effects of alcohol exposure and withdrawal on structural alterations in pyramidal neurons in two prefrontal cortical regions implicated in addiction, the agranular insular cortex (AID) and prelimbic cortex (PrL; Zilles’ area Cg3, [15]). These regions display complementary changes in response to drug exposure and other forms of experience, such as stress [16,17,18], and are connected to the nucleus accumbens (NAc) core and shell, which have been implicated in drug seeking, reward learning, and reinforcement [19,20,21,22,23,24,25]. The NAc core receives prominent afferents from AID and PrL [26,27], and the NAc shell innervates the ventromedial ventral pallidum [28,29,30,31]. This pallidal region projects to the mediodorsal nucleus of the thalamus [32], which has strong reciprocal connections with PrL and AID [33]. 

NAc shell and core medium spiny neurons (MSNs) undergo distinct structural modification following alcohol exposure and withdrawal. Peterson, et al. [1], using the same exposure protocol used in the present study in the rat, reported that chronic intermittent exposure (CIE) to vaporized ethanol for 10 days followed by cessation yielded distinct patterns of neuronal morphological alterations in NAc shell and core MSNs. At one day following cessation, MSNs of the core and shell displayed modest reductions in dendritic branching and length. At seven days post-cessation, the dendritic length and branching of shell MSNs remained reduced, whereas core MSNs displayed increases that matched or surpassed those observed in non-exposed rats. Mature spines were increased in the core at 24 hours post-cessation, and at seven days post-cessation the density of less mature spines was increased in both regions. Zhou, et al. [34] also observed differential effects of alcohol exposure and withdrawal on NAc core and shell spine density in alcohol-preferring rats that had continuous access to alcohol compared to rats that underwent repeated bouts of alcohol deprivation. Spine density in the NAc core and shell MSNs was reduced in the continuous access group, whereas repeated deprivation yielded reductions in spine density exclusively in the NAc shell. Spiga, et al. [2] exposed rats to ethanol for 20 days and demonstrated decreased thin spine density in the NAc shell exclusively in animals that were in withdrawal from alcohol; no effects of ethanol exposure were observed immediately following cessation. Uys, et al. [35] exposed adult mice to a vaporized ethanol in a CIE protocol (16 hours for four days per week for four weeks), which yielded increased spine density in the NAc core at 0 h (no withdrawal) that reverted back to control levels by 72 hours, with the exception of an increase in mature (mushroom) spines. Collectively, these observations indicate differential effects of ethanol exposure and withdrawal in the NAc core and shell, with variations in morphological changes due to multiple factors, such as exposure paradigms, species, and the timing of measurements. 

Previous research has established that drugs of abuse significantly alter the neurobiology of the NAc and frontal cortex. These findings motivated the present study to investigate the implications of ethanol exposure and withdrawal on dendritic morphology in AID and PrL, based on direct and indirect connectivity with the NAc core and shell. Furthermore, Varodayan, et al. [36] recently observed increased mature dendritic spines in layer II/III PrL neurons at six–seven days post-cessation, following seven weeks of CIE to ethanol. No effects were observed in layer V PrL neurons or in infralimbic neurons. Considering the findings of Peterson, et al. [1] in NAc with a considerably shorter CIE period, we were motivated to evaluate effects in PrL and AID following a comparatively shorter CIE duration. Dendritic length, branching, and spine density in AID and PrL were quantified using the same tissue utilized in the study of Peterson, et al. [1]. Adult male Sprague–Dawley rats were passively exposed to vaporized ethanol (~37 mg/L; 12 h/day) or air (control) for ten consecutive days. This pattern of ethanol exposure yields blood ethanol concentrations ranging from 150–200 mg/dL (0.15–0.20%), which produces robust physical dependence and increases anxiety-like behaviors [37] and significant alterations in glutamatergic and GABAergic neurotransmission in the amygdala [38,39]. Following the withdrawal period (one or seven days) or control exposure, the brains were extracted for Golgi–Cox staining [40] and the dendritic length, branching, total spine density, and density of specific spine types on layer II/III pyramidal neurons of AID and PrL were quantified. The relationship between dendritic changes in the frontal cortical regions quantified here and dendritic modifications in the shell and core of the NAc from the Peterson, et al. [1] study were also analyzed.

## 2. Materials and Methods

### 2.1. Subjects 

Male Sprague–Dawley rats (120–140 g) were obtained from Harlan Laboratories at the beginning of the experiments described in the following section. A total of 25 rats were assigned to one of three conditions described below. All animals were group-housed in an animal care facility at 23 °C with a 12 h light/dark cycle and given food and water ad libitum. Rats were weighed daily to ensure that at least 80% of their free-feeding weight was maintained during vapor chamber ethanol exposure. All animal procedures were performed in accordance with protocols approved by the Wake Forest School of Medicine Animal Care and Use Committee (protocol #A16-104) and were consistent with the National Institutes of Health Guide for the Care and Use of Laboratory Animals. Brain tissue was transferred to the University of New Mexico Main Campus for analysis, as approved by the Institutional Animal Care and Use Committee (protocol #100765). 

### 2.2. Chronic Ethanol Exposure and Withdrawal

Ethanol exposure was accomplished via an ethanol vapor chamber similar to that used in other studies [41]. Briefly, animals in their home cages were placed into airtight Plexiglas enclosures and exposed to either ethanol vapor (~37 mg/L) or room air (Con) during the light cycle (12 h/day) for 10 consecutive days. Tail blood was taken from sentinel animals periodically during the exposure to monitor blood ethanol concentration. This exposure produces blood-ethanol concentrations in the range of 150–200 mg/dL. Animals were then euthanized with an overdose of sodium pentobarbital followed by a transcardial perfusion (0.9% saline), resulting in exsanguination either one day (precisely 24 hours) or seven days after the last ethanol exposure. Control animals were euthanized two or three days after the final exposure to air. Whole brains were extracted for Golgi–Cox staining and analysis. 

### 2.3. Golgi–Cox Staining and Analysis

Brains were immersed in Golgi–Cox solution [42] for 14 days and subsequently immersed in 30% (*w*/*v*) sucrose for at least three days. The brains were then cut in coronal sections (200 μm thick) on a vibrating microtome, mounted on slides subbed in 2% gelatin, and stained according to the procedures described by [40]. 

**Figure 1:**
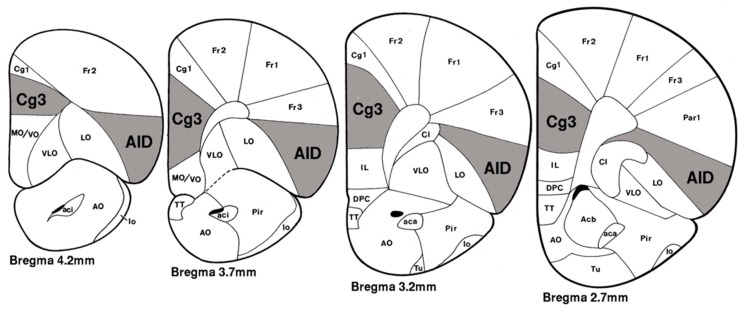
Agranular insular cortex (AID) and Prelimbic (Zilles’ area Cg3) regions extending from 2.7 to 4.2 mm anterior to Bregma from which neurons were sampled (Adapted from Zilles [15]). Abbreviations for other areas shown here, but not analyzed in the present study: Acb, nucleus accumbens; aci, anterior commisure (intrabulbar); AO, anterior olfactory nucleus; Cg1, anterior cingulate, Cl, claustrum; DPC, dorsal peduncular cortex; Fr1–3, frontal cortex; IL, infralimbic cortex; lo, lateral olfactory tract; LO, lateral orbital area; MO, medial orbital area; Par1, parietal cortex; Pir, piriform cortex; TT, taenia tecta; Tu, olfactory tubercle; VLO, ventrolateral orbital area; VO, ventral orbital area. Reprinted from [43].

Pyramidal neurons from layer II/III of the prelimbic cortex (PrL; Zilles’ area Cg3) and agranular insular cortex (AID) were selected for analysis (see Figure 1). The brain regions of interest were first identified at low power (200× magnification), and neurons were traced at 500× (final magnification) using the camera lucida technique on an Olympus light microscope (Model BX51) equipped with a drawing attachment. Sampling for all regions included sections ranging from 2.7 to 4.2 mm anterior to Bregma. Selection was limited to neurons that were not obscured by stain precipitate, blood vessels, astrocytes, or other artifacts, and had intact dendritic fields that were well impregnated and visible within a single section. All tracings were performed by an experimenter who was blind to exposure conditions. Ten neurons were drawn for each region of interest (five per hemisphere) for each animal, totaling twenty samples per brain. Outcome measures were averaged for each rat, collapsing across hemispheres.

Dendritic branching was measured by counting bifurcations for each dendrite [44]. Dendritic segments prior to the first bifurcation from the soma were designated as first-order branches in the basilar field and the primary apical dendrite was considered order 0. Branch order was incremented by one for each subsequent bifurcation on a given dendritic branch. The number of first- through sixth-order (and higher) branches were quantified and an estimate of total branches was determined from these values. Dendritic length was measured using a Sholl analysis of ring intersections [45]. A series of concentric rings at 20 μm increments (calibrated to the final magnification of 500×) printed on a transparency was centered over the soma. The total number of intersections between each ring and dendritic branches was counted and converted to estimates of dendritic length as a function of distance from the soma (i.e., for each 20 μm segment) separately for the apical and basilar fields. Overall (total) dendritic length was obtained by summing the apical and basilar measures.

Spine density was measured by tracing terminal tips (>40 μm in length) of third-order branches or higher at high power (2500× final magnification). Spines along the sampled dendritic segment were characterized by maturation state: filopodial, thin, stubby, mushroom, multi-headed mushroom, and other multi-headed. A total of ten segments were sampled per region (PrL and AID). Selected samples were unobstructed by other dendrites, glial cells, blood vessels, or other artifacts. 

### 2.4. Statistical Analyses 

Measures of total dendritic length, branching, and spine density were analyzed in separate one-way analyses of variance (ANOVAs) with group (Control, 24 h withdrawal, and seven day withdrawal) as a between-subjects factor. Significant main effects were followed by Tukey’s honestly significant difference (HSD) tests. Additional one-way ANOVAs were conducted separately for apical and basilar dendritic field measures to further characterize the effects of ethanol exposure and withdrawal on dendritic length, branching, and spine density. For dendritic length, repeated measures ANOVAs (RM-ANOVAs) were conducted with distance from the soma (averaged for seven segments, 40 µm each) as a within-subjects factor. For branch counts, repeated measures ANOVAs were conducted with branch order (1–6+) as a within-subjects factor. Geisser–Greenhouse correction was applied for cases in which violations of sphericity were observed. For spine morphology, separate ANOVAs for each spine morphology were conducted for the total cell, apical field, and basilar field, with significant main effects followed by Tukey’s HSD post-hoc tests. To evaluate relationships between alcohol-related dendritic morphology and spine changes in the NAc core/shell [1] and frontal cortex regions presented here, ANOVAs were conducted with region as a within-subjects factor. Partial eta squared ($\eta_{p}^{2})$ or Cohen’s d are reported for all effects.

## 3. Results

**Figure 2:**
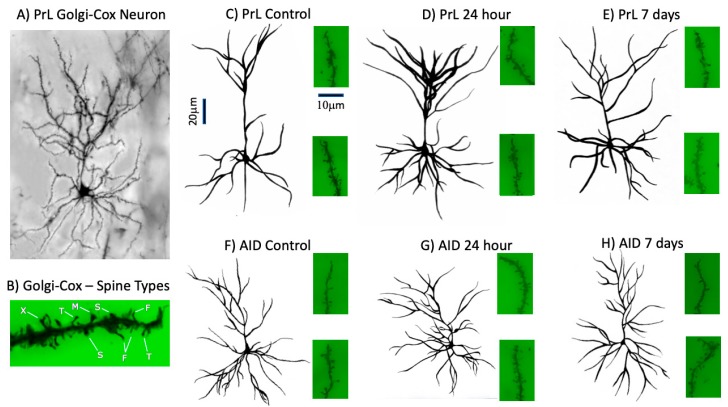
Representative Golgi–Cox staining of a layer II/III PrL neuron (**A**) and terminal spine segment (**B**), illustrating the quality of the staining. Example spine types (T = thin, S = Stubby, M = Mushroom, F = Filopodial, X = multi-headed) are shown in B. Representative camera lucida drawings of layer II/III neurons and images of spine segments from the apical and basilar fields are shown in (**C**,**D**) (PrL) and (**F**,**G**) (AID). The vertical scale bar (20 μm) provides scale for neurons in panels (**A**) and (**C**–**H**). The horizontal scale bar (10 μm) provides scale for spine segments in panels (**C**–**H**).

Representative images of the Golgi–Cox staining of neurons and spines, camera lucida drawings, and images of spine segments are shown in Figure 2. 

### 3.1. Total Dendritic Length, Branching, and Spine Density in PrL and AID

**Figure 3:**
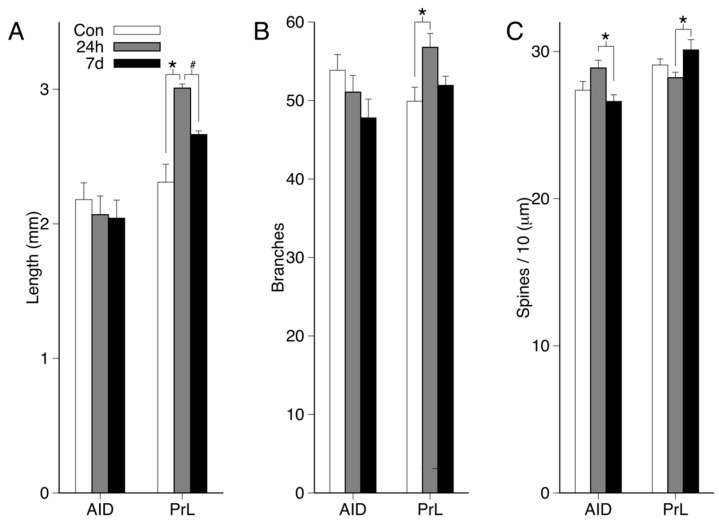
Mean + standard error of the mean (SEM) dendritic length (**A**), branch counts (**B**), and spine density (**C**) for AID and PrL neurons in each group. Values include basilar and apical dendritic fields. Figure 3, Figure 4 and Figure 5 provide values separately for the basilar and apical dendritic fields (*, *p* < 0.05, #, *p* = 0.052).

Mean dendritic length, branching, and spine density are shown in Figure 3A–C. There were no significant group main effects for dendritic length or branching in AID (all *p*s > 0.16). There were significant group main effects in PrL for dendritic length (*F*(2,21) = 12.25; *p* < 0.001; $\eta_{p}^{2}$ = 0.538) and branching (*F*(2,21) = 4.96; *p* = 0.017; $\eta_{p}^{2}$ = 0.321). Tukey’s HSD tests revealed that, compared to controls, the 24 h group had greater dendritic length (*p* < 0.001, d = 0.68) and branching (*p* = 0.015, d = 0.48). Although the 24 h group displayed higher dendritic length and branching values compared to the seven-day (7 d) group, these differences were not significant (both *p*s > 0.06). For dendritic length in PrL, post-hoc tests comparing the 7 d and control groups approached significance (7 d > control, *p* = 0.051, d = 0.56). 

There was a significant group main effect in AID for spine density (*F*(2,21) = 10.73; *p* = 0.018; $\eta_{p}^{2}$ = 0.318). Post-hoc tests revealed that the seven-day group had lower spine density than the 24 h group (*p* = 0.015, d = 0.48). Although inspection of the means (see Figure 2C) suggests that AID spine density was increased in the 24 h group, both the 24 h and 7 d groups did not significantly differ from controls (both *p*s > 0.12). For PrL, the group main effect approached significance (*F*(2,21) = 3.41; *p* = 0.052; $\eta_{p}^{2}$ = 0.245), which is primarily attributable to the increased spine density in the 7 d group relative to the 24 h group (7 d > 24 h, *p* = 0.049, d = 0.46).

### 3.2. Dendritic Length and Branching in Apical and Basilar Dendritic Fields of PrL and AID

**Figure 4:**
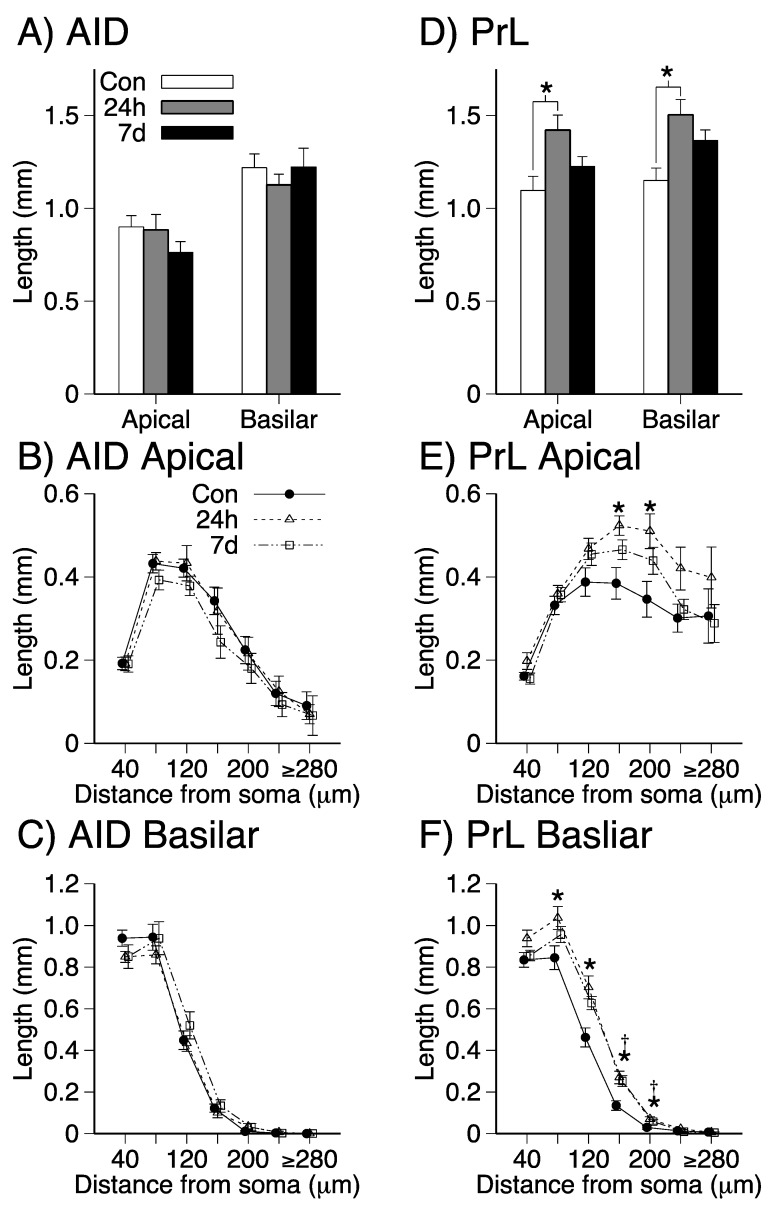
AID (**A**–**C**) mean (+SEM) dendritic length in apical and basilar fields (**A**), mean (±SEM) dendritic length as a function of distance from the soma for apical (**B**) and basilar (**C**) fields; PrL (**D**–**F**) mean (+SEM) dendritic length in apical and basilar fields (**D**), mean (±SEM) dendritic length as a function of distance from the soma for apical (**E**) and basilar (**F**) fields; (*, 24 h > Control, *p* < 0.05; † 7 d > Control, *p* < 0.05).

Mean estimates of total dendritic length and length as a function of distance from the soma in the apical and basilar dendritic fields of AID and PrL are shown in Figure 4. Group main effects for AID (Figure 4A) were not significant (all *p*s > 0.32). There were significant effects of group on dendritic length in the apical (*F*(2,21) = 5.340; *p* = 0.013; $\eta_{p}^{2}$ = 0.337) and basilar (*F*(2,21) = 6.562; *p* = 0.006; $\eta_{p}^{2}$ = 0.385) dendritic fields of PrL (Figure 4D). Post-hoc analyses revealed significant increases in PrL dendritic length in the 24 h withdrawal group compared to the control group in both dendritic fields (apical: 24 h > control, *p* = 0.010, d = 0.54; basilar: 24 h > control, *p* = 0.005, d = 0.64). For the PrL basilar field (Figure 4F), repeated measures ANOVAs with distance from soma as a within-subjects factor revealed significant interactions between group and distance from soma (Geisser–Greenhouse corrected *F*(3.289,120) = 3.83; *p* < 0.001; $\eta_{p}^{2}$ = 0.267). There were significant group effects for segments 80 µm, 120 µm, 160 µm, 200 µm from the soma (*p*s = 0.003–0.047; $\eta_{p}^{2}$s = 0.253–0.419). The 24 h group had higher dendritic length than the control group for each of these segments (*p*s = 0.003–0.041; ds = 0.33–0.63). The 7 d group had higher dendritic length than the control group for segments 160 µm and 200 µm (*p*s = 0.013–0.044; ds = 0.45–0.63). The group main effects for segments 40 µm, 240 µm, 280 µm from the soma in PrL basilar fields were not significant (*p*s > 0.092). All other interactions for AID (Figure 4B,C) and PrL (Figure 4E) were not significant (all *p*s > 0.32). Inspection of Figure 4E suggests that the observed increase in PrL apical dendritic length in the 24 h group compared to controls is due to increased dendritic length in the segments 160 µm and 200 µm from the soma (*p*s = 0.008–0.021; ds = 0. 45–0.46). 

**Figure 5:**
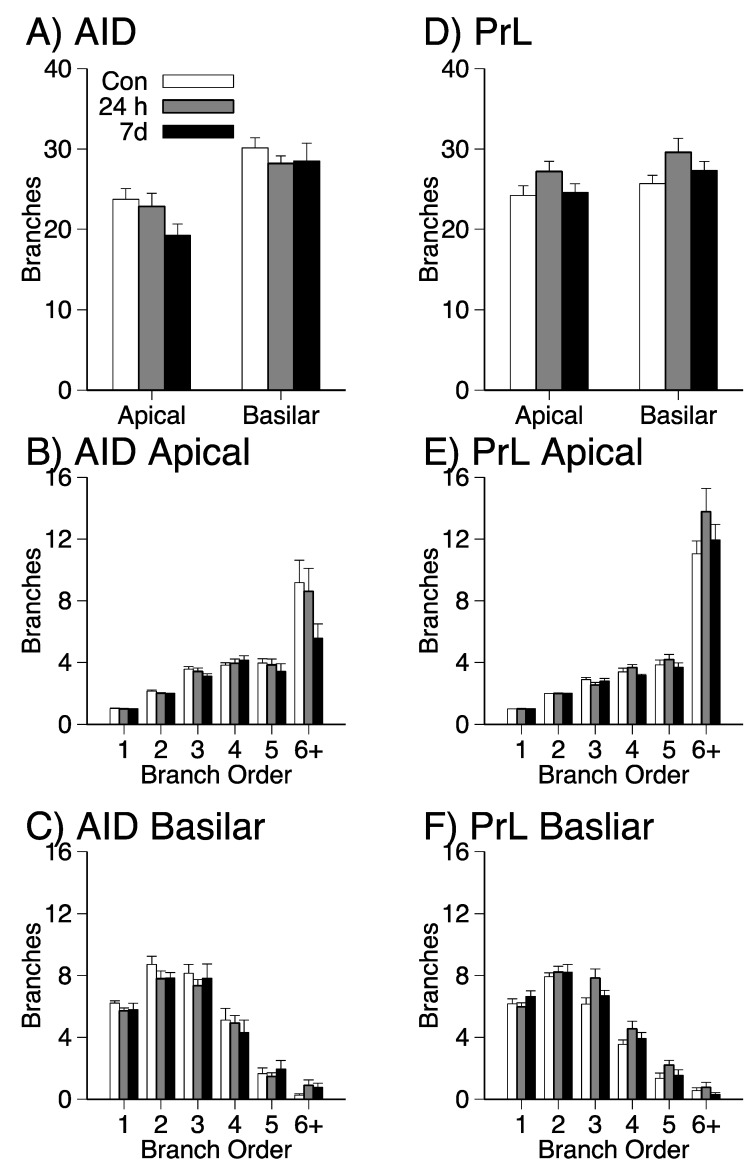
AID (**A**–**C**) mean (+SEM) branch counts in apical and basilar fields (A), mean (±SEM) branch counts for each branch order for apical (**B**) and basilar (**C**) fields; PrL (**D**–**F**) mean (+SEM) branch counts in apical and basilar fields (**D**), mean (±SEM) branch counts for each branch order for apical (**E**) and basilar (**F**) fields.

Mean branch counts in the apical and basilar dendritic fields of AID and PrL for each group are shown in Figure 5. There were no significant group effects for mean branch counts (Figure 5A,D; all *p*s > 0.09). Separate repeated measures ANOVAs with group and branch order (1–6+) also failed to detect significant interactions (Figure 5B,C,E,F; all *p*s > 0.06).

### 3.3. Dendritic Spine Density in Apical and Basilar Fields of PrL and AID

**Figure 6:**
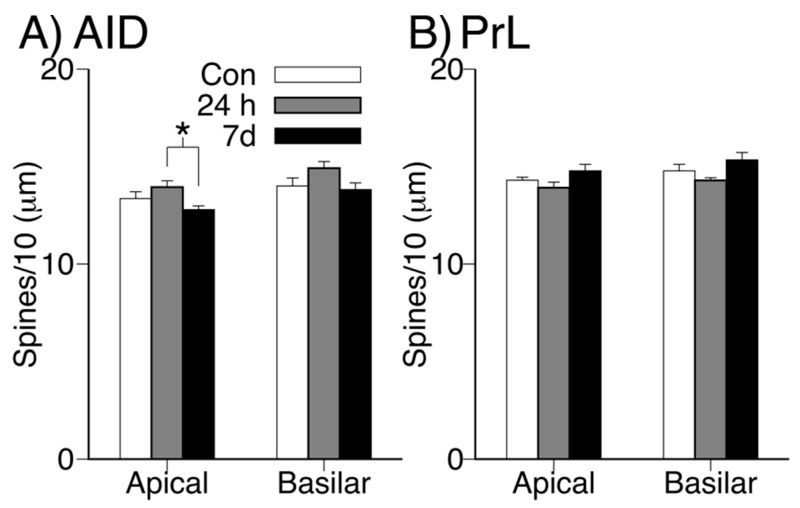
Mean (+SEM) spine density in apical and basilar dendritic fields in AID (**A**) and PrL (**B**). (* *p* < 0.05).

Mean total apical and basilar dendritic spine density (spines/10 μm) in AID and PrL for each group is shown in Figure 6. There was a significant main effect of alcohol withdrawal on total spine density in the apical dendritic field of AID neurons (*F*(2,21) = 3.761; *p* = 0.040; $\eta_{p}^{2}$= 0.264). Post-hoc analyses revealed that this effect was attributable to a significant difference between the 24 h withdrawal group and the 7 d withdrawal group (24 h > 7 d; *p* = 0.031, d = 0.33). Although there was a similar numerical pattern in the basilar field of AID neurons, this effect was not significant (*p*s > 0.09). There were no significant effects of group on total spine density in either dendritic field in PrL (all *p*s > 0.05). 

### 3.4. Spine Morphology in PrL and AID

Mean spine density (spines/10 μm) for each spine morphology (filopodial, thin, stubby, cup-shaped, mushroom, multi-headed, and multi-headed mushroom) for the total cell, apical field, and basilar field in AID and PrL are shown in Table 1. In AID, there were main effects of group for thin spines (*F*(2,21) = 4.155; *p* = 0.03; $\eta_{p}^{2}\;$= 0.284) and multi-headed mushroom spines (*F*(2,21) = 3.461; *p* = 0.05; $\eta_{p}^{2}$ = 0.248) (all other *p*s > 0.14). The group effect for thin spines was also observed in the apical dendritic field (*F*(2,21) = 3.715; *p* = 0.042; $\eta_{p}^{2}$ = 0.261) and approached significance in the basilar dendritic field (*F*(2,21) = 3.42; *p* = 0.052; $\eta_{p}^{2}\;$= 0.246). Inspection of the group means suggests that, relative to controls, the density of thin spines increased in the 24 h group and numerically decreased in the 7 d group. Post-hoc analyses revealed that the 24 h group had significantly higher thin spine density than the 7 d group for the total cell (*p* = 0.025, d = 0.31) as well as in the apical dendritic field (*p* = 0.033, d = 0.33), with a similar trend in the basilar field (*p* = 0.059, d = 0.30). All other group comparisons for thin spines were non-significant (all *p*s > 0.178). Separate analysis for each dendritic subfield revealed no main effects of group for multi-headed mushroom spines (*p*s > 0.095). Though group main effects for the entire cell were not observed for cup-shaped spines, separate analyses within dendritic subfields revealed a group main effect for the density of cup-shaped spines in the apical field (*F*(2,21) = 4.838; *p* = 0.019; $\eta_{p}^{2}$ = 0.315). Post-hoc analysis revealed that the 7 d group had a higher density of cup-shaped spines compared to controls (*p* = 0.04, d = 0.03) and the 24 h group (*p* = 0.007, d = 0.04). There were no effects of alcohol withdrawal on spine types in the basilar dendritic field of AID [15]. There was an effect of alcohol withdrawal on the number of mushroom-shaped spines in the apical dendritic field of PrL (*F*(2,21) = 11.817; *p* < 0.001; $\eta_{p}^{2}$ = 0.530), which was due to significant differences between the 7 d group and both the control and 24 h withdrawal groups (7 d < control & 24 h; *p*s < 0.002, ds =0.11–0.12). In the basilar dendritic field of PrL, there was a significant withdrawal effect on the number of stubby spines (*F*(2,21) = 4.428; *p* = 0.025; $\eta_{p}^{2}$ = 0.297), which was driven by a significant effect in the control group compared to both the 24 h and 7 d withdrawal groups (*p*s < 0.030, d = 0.05–0.06]. 

### 3.5. Relationship between Nucleus Accumbens and Frontal Cortex Dendritic Morphology and Spine Density Following Ethanol Exposure and Withdrawal 

NAc core and shell dendritic morphology and spine density data were previously published (see ref. [1]) and are summarized in Table A1. The key findings from [1] were decreases in length and branching but increased spine density in the shell and core in the 24 h group. In the 7 d group, length and branching returned to or exceeded control levels in the core, whereas these measures remained decreased and comparable to the 24 h group in the shell. Spine density began to decrease toward control levels in the 7 d group in the core and shell. Peterson et al. [1] reported that only stubby and mushroom spine types displayed changes in the 24 h and 7 d groups relative to controls (see Table A1). Significant increases were observed in stubby spines in the 7 d group in both regions, and an increased density of mushroom spines in the core in the 24 h group. The pattern observed in PrL in the present study is opposite to that observed in the NAc, particularly with respect to the observations in the core. We note that there were no significant correlations among measures in the NAc and frontal cortex (all *p*s > 0.14). The statistical outcomes most relevant to understanding these apparent opposing relationships between alcohol-related changes in NAc and frontal cortex, however, are group × region interactions and follow-up tests conducted with region as a within-subjects factor. For these analyses, all values were converted to percentage of control group means to allow for analyses of interactions independently of main effects of region. The observation of different outcomes in the apical and basilar fields also motivated separate analyses for each field rather than the use of measures for the entire cell. 

There were significant group × region interactions for dendritic length observed for the PrL apical field and core (*F*(2,21) = 6.45; *p* = 0.007; $\eta_{p}^{2}$ = 0.381), and the PrL basilar field and core (*F*(2,21) = 5.53; *p* = 0.012; $\eta_{p}^{2}\;$= 0.345). Follow-up analyses confirmed that interaction was driven by the distinct changes in the 24 h groups observed in PrL and the core (PrL > core; *p*s < 0.008; $\eta_{p}^{2}$s > 0.66). Similarly, there were significant group × region interactions for dendritic length observed for the PrL apical field and shell (F(2,21) = 6.45; *p* = 0.007; $\eta_{p}^{2}$ = 0.381), and the PrL basilar field and shell (*F*(2,21) = 5.53; *p* = 0.012; $\eta_{p}^{2}\;$= 0.345). Follow-up analyses confirmed that interaction was driven by distinct changes in the 24 h groups observed in PrL and the shell (PrL > shell; *p*s < 0.006; $\eta_{p}^{2}$s > 0.68), as well as region differences in the 7 d group for the PrL basilar field (PrL > shell; *p* = 0.011; $\eta_{p}^{2}$ = 0.63). There were no significant group × region interactions for branching or total spine density for PrL and the core or shell (all *p*s > 0.057), and no interactions for AID and the NAc for any measures (all *p*s > 0.07).

There were no significant correlations between mushroom or stubby spine densities in the NAc or PrL (all *p*s > 0.16). There were, however, significant interactions observed for mushroom spines in the core and PrL apical field (*F*(2,21) = 10.98; *p* = 0.001; $\eta_{p}^{2}$ = 0.511) and for the shell and the PrL apical field (*F*(2,21) = 10.65; *p* = 0.001; $\eta_{p}^{2}$ = 0.504). In both cases, the density of mushroom spines was increased in the PrL apical field but decreased in the core and shell in the 24 h group, whereas the opposite pattern was observed for the 7 d group. Follow-up analyses confirmed a significant decrease in the PrL apical mushroom spines relative to the core (*p* = 0.035; $\eta_{p}^{2}$ = 0.494), however, the difference was non-significant for the shell (*p* = 0.077; $\eta_{p}^{2}$ = 0.38). In the 7d group, the PrL apical mushroom spine density was increased relative to the core (*p* = 0.019; $\eta_{p}^{2}$ = 0.568) and shell (*p* = 0.012; $\eta_{p}^{2}$ = 0.619).

## 4. Discussion

**Figure 7:**
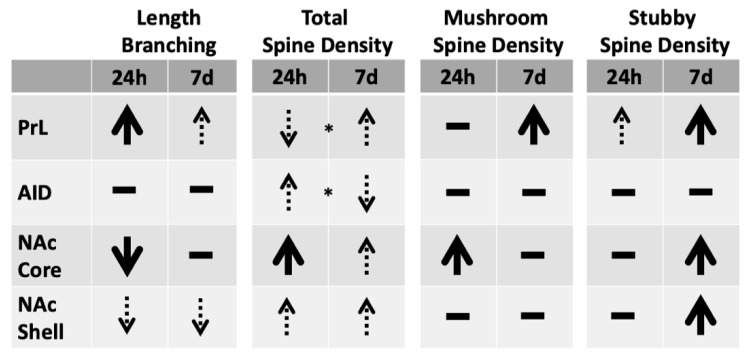
Summary of major findings from the present study and those reported in [1] for the 24 h and 7 d groups (relative to control). Significant modifications in dendritic length and spine density are indicated by large solid arrows. Non-significant trends are indicated in dotted lines. In some cases (e.g., total spine density for PrL) dotted arrows marked by * are included to reflect numerical trends from the control condition and significant differences between the 24 h and 7 d groups. Horizontal bars indicate no change from control.

CIE to ethanol followed by withdrawal produced significant alternations in dendritic morphology and spine density in the frontal cortex. In the prelimbic cortex (PrL), dendritic length and branching were increased at 24 h and reverted back to control levels at seven days post-cessation, whereas increased density of mature mushroom-shaped spines and less mature stubby spine types were observed at seven days post-cessation. The increased dendritic length in PrL at 24 h was observed in apical (160–200 μm from the soma) and basilar fields (80–200 μm from the soma), however, the increased branching was only significant for the total cell measures. Mushroom-shaped spines primarily increased in the apical field of PrL cells, whereas stubby spines increased in the basilar field. In AID there were transient (24 h group only) increases in spine density, which were primarily due to increases in thin spines of the apical field, in the absence of changes in overall dendritic length or branching. Overall, these observations indicate that PrL undergoes more prominent alterations in response to CIE to ethanol and withdrawal than AID, with transient changes in overall length and branching, followed by changes in dendritic spine density later during withdrawal. Comparison of the data from PrL with previously published results from the NAc core and shell [1] revealed complementary changes in dendritic length, branching, and spine density. The major patterns of CIE-related modifications in dendritic length, branching, and spine density for the present study and from [1] are summarized in Figure 7. At 24 h, there were decreases in length and branching in NAc core and shell MSNs, whereas PrL neurons underwent increases in these measures. At 7 d post-cessation, shell MSNs remained decreased, core MSNs returned to or exceeded control values, and PrL neurons reverted back to control levels. Thus, an opposite pattern of changes during withdrawal was observed in the NAc core and PrL from 24 h to 7 d post-cessation. Furthermore, mushroom-shaped spines were increased only at 24 h in the core and shell, but were increased at 7 d in PrL. Collectively, these observations dissociate short- and long-term changes across regions and indicate that long-term alterations in prelimbic cortex neurons during withdrawal involve increases in spine density, whereas long-term changes in NAc primarily involve decreases in the length of dendrites in the NAc shell. 

Consistent with our finding of increased spine density in AID, alcohol exposure results in increased dendritic spine density in the lateral orbital frontal cortex of mice [5], an area adjacent to AID. We note that there were decreases in multi-headed spines in AID at 24 h and 7 d, however, due to the overall low frequency of these spine types (<1 per 50 μm), this finding should be considered with caution. Also consistent with the present findings, Varodayan, et al. [36] reported increased mature spines in layer II/III prelimbic neurons at six–seven days post-cessation. No effects were observed in layer V prelimbic neurons or in infralimbic neurons. These authors used a much longer exposure period (seven weeks) than that utilized in the present study (10 days). Therefore, long-term increases in mature spines appear to occur for various durations of exposure and can occur with as little as 10 days of CIE to ethanol. Future studies should identify the minimum duration of CIE to ethanol required to achieve these effects and examine whether other protocols of ethanol exposure yield similar outcomes. The results of the present study also suggest complementary changes between PrL neurons and hippocampal CA1 pyramidal cells. McMullen, et al. [4] observed reduced branching in CA1 of the hippocampus in the short term, followed by increased length in the long term, following cessation and deprivation from ethanol. Thus, multiple brain regions appear to display complementary structural changes in neurons to those observed in PrL following ethanol exposure and withdrawal.

After 7 d of ethanol withdrawal there were significant increases in spine density in PrL neurons relative to the 24 h group, which was largely due to increased mushroom spines. Because dendritic spines represent the primary sites of excitatory synaptic connections, these changes suggest an increase in the overall amount of excitatory synaptic space on PrL neurons at 7 d post-cessation. Mushroom-shaped spines are the most mature and stable type of spine, followed by stubby, thin, and filopodial [46,47], and are associated with high post-synaptic densities [48]. Mushroom-shaped spines also appear to be the exclusive site of the spine apparatus [49], which is implicated in synaptic plasticity [50]. This suggests that the changes observed in PrL may be relevant to withdrawal-related structural plasticity in comparatively stable dendritic spines. One possibility is that such changes impact excitatory neurotransmission. Ethanol exposure increases extracellular glutamate levels in the NAc [51,52] and enhances glutamatergic synaptic transmission in a number of brain regions [41,53,54,55]. Future studies should examine the relationship between withdrawal-related increases in mature spines and excitatory transmission. The functional properties of stubby spines are not well understood, however, synapse formation occurs less frequently on stubby spines compared to mushroom spines [56,57], and the lack of a distinctive head has been taken to indicate reduced compartmentalization of Ca^2+^ signaling from the dendrite [58]. Increases in brain-derived neurotrophic factor (BDNF) in the dorsal striatum have been observed following ethanol exposure [59,60], which has been proposed to contribute to a homeostatic process that breaks down with extensive exposure [61]. BDNF exposure is also associated with increases in stubby spines [62], suggesting a link between ethanol exposure, BDNF, and alterations in the expression of stubby spines. Future research is needed to investigate these relationships in the frontal cortex regions examined here.

Understanding the functional and behavioral consequences of the dendritic changes observed in AID and PrL will also require additional research. Considering the pattern of changes reported here, the transient changes (24 h group only) observed in PrL and AID may contribute to the broader range of behavioral and cognitive changes associated with short term withdrawal or deprivation. Of course, in addition to involvement in reinforcement and reward processes, the prefrontal cortex is involved in a wide variety of functions, including memory and decision making [63], predictive fear learning [64], coping and resilience [65], and emotion regulation [66]. AID also plays a critical role in social behavior and behavioral flexibility [67], anticipatory behaviors, and pain response behaviors [68,69]. All of these behavioral and cognitive processes could be related to structural plasticity in the prelimbic and agranular insular cortex following ethanol exposure and withdrawal, and could contribute to behaviors observed during withdrawal. 

The findings from the present study should be considered in the context of several aspects of the design and analyses that place limitations on generalization. Interpretation of the results for the 24 h withdrawal group is not straightforward, as disentangling whether the outcomes reflect the direct effects of CIE to ethanol, withdrawal, or a combination of both is not possible in the study design utilized here (see also, [34]). We note that blood ethanol levels are at 0 only 2–3 h after the final exposure, and acute withdrawal somatic symptoms peak around 12–14 h and are no longer present by 24 h. Furthermore, CIE-induced increases in N-methyl-D-aspartate (NMDA) receptor synaptic function in the amygdala are expressed only after 24 h withdrawal and are not present at the end of the CIE period if animals are still intoxicated [70]. These outcomes could reflect distinct neurobiological processes unique to short-term and long-term withdrawal, and future studies to evaluate dendritic morphology and spine density immediately after ethanol is removed are needed to address this issue. Furthermore, the present study did not examine dendritic morphology changes following periods of withdrawal longer than seven days. The observations of increased mature spines at this duration suggests that the effects may persist, however, additional studies are needed to address this possibility. An examination of changes at intermediate time points (one–six days of withdrawal) is also needed to establish the earliest time point following cessation at which increases in mature spines can be detected. Passive exposure to ethanol, as used in the present study, has repeatedly yielded behavioral signs of withdrawal and physical dependence [37,71,72], as well as increased ethanol seeking/consumption [73,74] and anxiety [37,75,76]. It is, therefore, important to recognize that the morphological outcomes reported here could be related to behavioral alterations. Behavioral outcomes were not assessed in the present study to avoid complicating the interpretation of the results, because experience during behavioral testing could influence dendritic morphology and spine density and possibly interact with alcohol exposure and withdrawal. Future studies should directly evaluate relationships between structural changes in neurons and behavioral expressions of withdrawal. The exposure methods were also limited to passive vaporized ethanol exposure; thus, the present findings cannot address whether similar effects would be observed with voluntary consumption or are found only following physical dependence. We also note that the cells from the NAc and frontal cortex were analyzed by different experimenters (authors VLP and MEF, respectively). All analyses were performed blind to group membership, and values were converted to percentage of control values for comparisons across regions (and experimenters), thus, any variation between experimenters should be constant and not impact the analysis of interactions presented in Section 3.5. In the present study, all analyses were performed in layer II/III cells in PrL and AID to support the direct comparison of findings from PrL and AID, and in consideration of the recent findings following CIE by Varodayan et al., [36] in layer II/III neurons, but not layer V, discussed previously. Although we observed relationships between CIE-related changes in layer II/III neurons of PrL and MSNs in the NAc, the efferents from PrL to NAc emerge from layer V neurons, thus, it will be important for future studies to examine relationships between PrL and NAc for layer V neurons, as well as the superficial layers examined here. The present study was also performed in adult male rats. Future studies will be needed to determine if the outcomes observed here generalize to female rats. 

Several additional questions for future research are motivated by the present observations. Perhaps the most prominent of these is to determine the physiological and related behavioral outcomes associated with the patterns of alterations in dendritic morphology and spine density that follow CIE to ethanol and withdrawal. Electrophysiological measurements of glutamatergic synaptic transmission in the basolateral nucleus of the amygdala have been reported following ethanol exposure and cessation [41], which may be related to anxiety and related outcomes [70,77]. Dendritic morphology alterations following CIE to ethanol have been observed in the infralimbic cortex, and linked to altered fear extinction learning [78]. Whether similar relationships between electrophysiological alterations in prelimbic neurons and behavioral outcomes exist should be investigated. Theta-band activity in the prelimbic region has also recently been linked to addiction [79], and future research should evaluate if alterations in theta activity are related to changes in spine density and other morphological changes during ethanol withdrawal. Alcohol exposure also causes changes in dendritic morphology dorsal striatum [80], and relationships to changes in ventral striatum and frontal cortex should be investigated.

## 5. Conclusions

In summary, chronic passive exposure to ethanol and withdrawal were associated with structural alterations on layer II/III neurons of the prelimbic cortex, and, to a lesser extent, the agranular insular cortex. The most robust ethanol-related differences were the transient increases in dendritic length and branching in prelimbic neurons at 24 h post-cessation, and the increased mushroom-shaped spines at 7 d post-cessation. Transient changes in spine density in agranular insular cortex neurons were also noted. The results obtained in the prelimbic cortex are the opposite of those previously reported in the NAc core. The present data motivate future research to identify the functional and behavioral outcomes associated with these morphological alterations, which may be important for understanding the neural circuitry involved in ethanol exposure and withdrawal.

## Figures and Tables

**Table 1:** Mean (SEM) spine density (spines/10 μm) in AID (1A) and PrL (1B) for filopodial, thin, stubby, mushroom, cup-shaped, multi-headed, and multi-headed mushroom spines in each group. * *p* < 0.05, # *p* = 0.052. Total measures (averaged apical and basilar field data) are shown in bold text. Apical and basilar means are shown in italics. Grey shaded means indicate significant post-hoc comparisons (*p* < 0.05). For mushroom spines in the apical field of PrL post-hoc comparisons showed the 7 d group had significantly more spines than the control and 24 h groups, and controls had fewer stubby spines than both withdrawal groups.

**1.A. AID**			
	**Control**	**24h**	**7d**
**Filopodial**	**5.497 (0.132)**	**5.497 (0.198)**	**5.403 (0.135)**
Apical	*5.466 (0.151)*	*5.606 (0.273)*	*5.400 (0.157)*
Basilar	*5.528 (0.182)*	*5.388 (0.146)*	*5.406 (0.180)*
Thin *	**5.969 (0.279)**	**6.809 (0.456)**	**5.519 (0.156)**
Apical *	*5.984 (0.295)*	*6.491 (0.429)*	*5.306 (0.120)*
Basilar #	*5.953 (0.373)*	*7.128 (0.541)*	*5.731 (0.248)*
**Stubby**	**0.148 (0.020)**	**0.130 (0.017)**	**0.173 (0.044)**
Apical	*0.153 (0.025)*	*0.138 (0.030)*	*0.156 (0.038)*
Basilar	*0.144 (0.037)*	*0.122 (0.023)*	*0.191 (0.086)*
**Mushroom**	**0.311 (0.034)**	**0.283 (0.034)**	**0.373 (0.027)**
Apical	*0.250 (0.031)*	*0.266 (0.043)*	*0.388 (0.049)*
Basilar	*0.372 (0.047)*	*0.300 (0.029)*	*0.359 (0.033)*
**Cup-Shaped**	**0.102 (0.023)**	**0.125 (0.030)**	**0.153 (0.019)**
Apical	*0.088 (0.023)*	*0.059 (0.014)*	*0.163 (0.032)*
Basilar	*0.116 (0.032)*	*0.191 (0.061)*	*0.144 (0.025)*
**Multi-headed**	**1.514 (0.162)**	**1.544 (0.136)**	**1.634 (0.077)**
Apical	*1.322 (0.131)*	*1.338 (0.177)*	*1.322 (0.085)*
Basilar	*1.706 (0.275)*	*1.750 (0.137)*	*1.947 (0.141)*
Multi-headed mushroom *	**0.144 (0.044)**	**0.053 (0.012)**	**0.063 (0.007)**
Apical	*0.097 (0.037)*	*0.056 (0.014)*	*0.084 (0.012)*
Basilar	*0.191 (0.085)*	*0.050 (0.023)*	*0.041 (0.015)*
**1.B. PrL**			
	**Control**	**24h**	**7d**
**Filopodial**	**5.230 (0.378)**	**4.964 (0.469)**	**5.281 (0.227)**
Apical	*5.253 (0.148)*	*4.931 (0.174)*	*5.228 (0.103)*
Basilar	*5.206 (0.148)*	*4.997 (0.209)*	*5.334 (0.108)*
**Thin**	**6.255 (0.330)**	**6.234 (0.484)**	**6.139 (0.538)**
Apical	*6.131 (0.089)*	*6.244 (0.199)*	*5.994 (0.253)*
Basilar	*6.378 (0.167)*	*6.225 (0.196)*	*6.284 (0.156)*
Stubby *	**0.183 (0.081)**	**0.292 (0.099)**	**0.322 (0.088)**
Apical	*0.206 (0.035)*	*0.294 (0.042)*	*0.328 (0.045)*
Basilar *	*0.159 (0.025)*	*0.291 (0.044)*	*0.316 (0.047)*
Mushroom *	**0.497 (0.099)**	**0.497 (0.094)**	**0.689 (0.167)**
Apical *	*0.481 (0.036)*	*0.459 (0.037)*	*0.772 (0.071)*
Basilar	*0.513 (0.082)*	*0.534 (0.052)*	*0.606 (0.063)*
**Cup-Shaped**	**0.177 (0.082)**	**0.123 (0.052)**	**0.181 (0.061)**
Apical	*0.153 (0.030)*	*0.122 (0.023)*	*0.172 (0.027)*
Basilar	*0.200 (0.047)*	*0.125 (0.029)*	*0.191 (0.045)*
**Multi-headed**	**2.081 (0.462)**	**1.905 (0.325)**	**2.245 (0.513)**
Apical	*1.956 (0.169)*	*1.772 (0.109)*	*2.094 (0.154)*
Basilar	*2.206 (0.198)*	*2.038 (0.141)*	*2.397 (0.218)*
**Multi-headed mushroom**	**0.119 (0.072)**	**0.094 (0.130)**	**0.200 (0.111)**
Apical	*0.122 (0.055)*	*0.103 (0.045)*	*0.188 (0.041)*
Basilar	*0.116 (0.042)*	*0.084 (0.050)*	*0.213 (0.082)*

## Appendix A

**Table A1:** Mean (SEM) branches, dendritic length, and spine density (spines/10 μm; total, mushroom, and stubby spines) in the nucleus accumbens (NAc) core and shell for the control, 24 h, and 7 d groups from [1]. Grey shaded means indicate significant post-hoc comparisons (*p* < 0.05). For stubby spines in each region the 7 d group had significantly greater means than the control and 24 h groups. For mushroom spines in the core the 24 h group had values greater than the control and 7 d groups. # shown for 7 d branching and length values indicate a significant post-hoc difference between regions (Core > Shell).

	Control	24 h	7 d
**NAc Core**			
Branches	34.92 (1.252)	32.150 (1.014)	# 35.463 (1.639)
Dendritic Length (mm)	1.319 (0.071)	1.278 (0.058)	# 1.456 (0.057)
Total Spine Density	8.660 (0.290)	9.715 (0.519)	9.304 (0.547)
Mushroom Spine Density	2.424 (0.161)	*3.123 (0.267)*	2.332 (0.300)
Stubby Spine Density	2.813 (0.321)	2.829 (0.245)	3.940 (0.311)
**NAc Shell**			
Branches	34.33 (1.002)	32.088 (1.189)	# 32.563 (1.566)
Dendritic Length (mm)	1.333 (0.051)	1.261 (0.055)	# 1.293 (0.056)
Total Spine Density	8.976 (0.383)	9.234 (0.497)	9.355 (0.570)
Mushroom Spine Density	2.589 (0.161)	2.881 (0.105)	2.299 (0.223)
Stubby Spine Density	2.916 (0.403)	3.002 (0.254)	3.911 (0.284)
